# The efficacy of injury screening for lower back pain in elite golfers

**DOI:** 10.4102/sajp.v79i1.1843

**Published:** 2023-02-09

**Authors:** Samantha-Lynn Quinn, Benita Olivier, Warrick McKinon

**Affiliations:** 1 Department of Physiotherapy, School of Therapeutic Sciences, Faculty of Health Sciences, University of the Witwatersrand, Johannesburg, South Africa; 2 Movement Physiology Research Laboratory, Faculty of Health Sciences, University of the Witwatersrand, Johannesburg, South Africa; 3 School of Medicine, Keele University, Keele, Staffordshire, United Kingdom

**Keywords:** golf, back, injury, prevention, lumbar, spine, rehabilitation, junior, sport, physiotherapy

## Abstract

**Background:**

Injury prevention is a growing focus for golfers in general and for elite golfers in particular. Movement screening has been proposed as a possible cost-effective means of identifying underlying risk factors and is widely utilised by therapists, trainers and coaches.

**Objectives:**

Our study aimed to establish whether results from movement screening were associated with subsequent lower back injury in elite golfers.

**Methods:**

Our prospective longitudinal cohort study with one baseline time point included 41 injury-free young elite male golfers who underwent movement screening. After this, the golfers were monitored for 6 months for lower back pain.

**Results:**

Seventeen golfers developed lower back pain (41%). Screening tests that were able to differentiate golfers who developed and those who did not develop lower back pain, included: rotational stability test on the non-dominant side (*p* = 0.01, effect size = 0.27), rotational stability test on the dominant side (*p* = 0.03; effect size = 0.29) and plank score (*p* = 0.03; effect size = 0.24). There were no differences observed in any other screening tests.

**Conclusion:**

Out of 30 screening tests, only three tests were able to identify golfers not at risk of developing lower back pain. All three of these tests had weak effect sizes.

**Clinical implications:**

Movement screening was not effective in identifying elite golfers at risk of lower back pain in our study.

## Introduction

Elite golfers have a moderate risk of injuries, the majority of which occur in the lower back (Robinson et al. 2019; Smith et al. 2018). Lower back pain prevalence among professional golfers is estimated to be around 50% (Smith et al. 2018). There has been increasing awareness regarding preventing lower back pain in elite golfers. Several studies have shown an association between certain swing kinematics and developing lower back pain (Smith et al. 2018). The tools used to assess swing kinematics are expensive and require advanced skills to utilise. Movement screening has been proposed as a possible cost-effective alternative to identifying underlying risk factors and is widely utilised by therapists, trainers and coaches in the golf industry (Titleist Performance Institute 2021). There is, however, conflicting evidence regarding the usefulness of movement screening in identifying athletes at risk of injury (Moran et al. 2017).

According to a systematic review by Bahr (2016), three essential steps are required to develop and validate a risk screening tool. The first step is to identify the strength of risk factor association with injury development (Bahr 2016). In support of this first step, several studies have shown an association between trunk and hip muscle performance in golfers and back pain (Smith et al. 2018). In particular, decreased *transverse abdominis* endurance, decreased trunk strength in all planes, reduced trunk rotational endurance towards the lead side, reduced isokinetic trunk extension and reduced isometric hip adduction have been associated with lower back pain in golfers (Smith et al. 2018). Asymmetry between the left- and right-side endurance during the side bridge is associated with the development of lower back pain (Evans et al. 2005), while pooled analysis of trunk extension range of motion did not correlate with developing lower back pain (Smith et al. 2018). Similarly, pooled analysis of lead and trail hip external rotation range of motion and internal rotation was also not associated with developing lower back pain (Smith et al. 2018). Side-to-side hip internal rotation range of motion asymmetry has been shown to be greater in golfers with lower back pain (Smith et al. 2018). These studies give theoretical support to the concept that movement screening tools may be able to identify golfers at risk of developing lower back pain.

The second step in developing a risk screening tool involves screening athletes with the same test, but in this second scenario, a predetermined cut-off score is used to separate athletes at risk of injury from the rest (Bahr 2016). The athletes should then be monitored for injury over a set period. Step three involves conducting a randomised controlled trial in which athletes at risk are identified using the risk screening tool and then randomly assigned to control or a treatment group (Bahr 2016). The treatment group is given an intervention aimed at addressing the risk factor identified by the tool. The athletes should then be monitored, and if injury incidence reduces in the treatment group, the screening tool can be validated for that patient group (Bahr 2016).

Risk screening tools are frequently used to identify golfers at risk of lower back pain, but their effectiveness in identifying athletes at risk is uncertain (Trinidad-Fernandez et al. 2019). Our study aimed to determine if commonly used screening tools were associated with developing lower back pain in young elite male golfers.

## Methods

**FIGURE 1:**
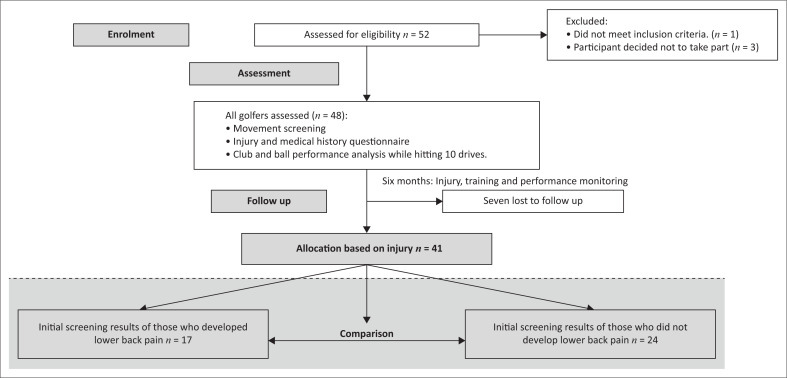
Flow diagram overview.

Our study employed a prospective longitudinal cohort design and had one baseline assessment and took place at an elite golf academy in Johannesburg. A flow diagram overview of our study is shown in Figure 1.

To prevent selection bias, all injury-free golfers at the golf academy, who were also classified as elite players by their coaches (All the coaches at the Golf School of Excellence were Professional Golfers’ Association (PGA) golf professionals or PGA golf apprentices), were invited to participate in our study. ‘Injury free’ was defined as not experiencing pain or discomfort while playing golf or performing activities. Further inclusion criteria included being male and between the ages of 16 and 30. As a result of limited access to female golfers, we were unable to focus our study on female golfers. It was also not possible to combine female golfers’ movement screen measurements with male golfers’ measurements as women golfers are likely to have reduced strength compared to male golfers (Ramos et al. 1998). Golfers under 18 years old (junior golfers) were included in the study; therefore, a handicap cut-off was not applied. Golfers with serious spinal or hip pathology were excluded. Data were collected from February 2017 to January 2018.

### Procedure

Each golfer underwent a baseline assessment. This baseline assessment included: a demographic questionnaire, a medical history questionnaire, previous injury questionnaire, anthropometric measurements, a movement screening and a swing performance assessment. As a result of the sensitive nature of the medical screening and injury questionnaire, golfers were permitted to omit certain questions or forgo this questionnaire and still take part in our study.

At the initial assessment, the golfers underwent a swing performance assessment. All participants were asked to hit the golf ball with their driver 10 times as they would during a competition where both distance and accuracy are important. Club and ball performance was assessed using the FlightScope X2 Elite system (FlightScope X2 Elite, FlightScope (Pty) Ltd., Stellenbosch, South Africa), a 3D Doppler tracking radar designed for golf-specific application (FlightScope 2021) as per Leach et al. (2017).

The initial assessment also included a movement screening. The movement screening was conducted by the first author and two physiotherapy research assistants (all three assessors are qualified physiotherapists). The first author also briefed the assessors as to how to conduct and score the tests. They were also provided with written explanations of the tests and scorings. The movement screen included the following tests (with their various sub-divisions), 30 in total: single leg squat, deep overhead squat; hurdle step, in line lunge, straight leg raise, trunk stability push-up; spinal extension test, rotational stability test, spinal flexion clearing test, pelvic tilt test, pelvic rotation test, torso rotation test, toe touch test, 90/90 test, single leg bridge, lower quarter rotation test, seated trunk rotation test, plank, sit-ups, push-ups and oblique sit-ups (Online Appendix 1, Section 1 contains descriptions of each of the tests). These tests were selected for their utility for screening golfers before and during the season for risk of injury (Titleist Performance Institute 2021). The scoring for each of the tests has been provided in Online Appendix 1, Section 2.

After the initial baseline assessment, the golfers were monitored for 6 months for injury, training and competition performance. Participants were asked to contact the research team immediately if any injury were sustained. Golfers were also contacted weekly to determine if they sustained an injury or not during the previous week. ‘Injury’ to the lower back was defined as ‘pain and discomfort that was localised below the costal margin and above the inferior gluteal folds, with or without leg pain’ (Burton et al. 2006). The injury did not need to affect golf participation. Training was monitored via a weekly training log, which was completed by the golfer. Golfer performance was ranked using the 2017 yearly order of merit score. Most golfers played in a weekly order of merit tournament, resulting in a yearly order of merit score.

### Data reduction and analysis

The FlightScope data were recorded as an average of 10 swing cycles for each participant. The FlightScope data recorded carry distance (metres), roll (metres), total distance (metres), ball distance (metres) and club head speed (km/h).

Two golfers were left-handed and played golf left-handed. For this reason, measurements were recorded as dominant and non-dominant.

### Statistical analysis

Data were analysed using Statistica^®^ Version 12 (StatSoft Inc, Tulsa, Oklahoma, United States). Categorical data (Online Appendix 1, Section 3) are presented as frequencies and analysed using Pearson’s chi-squared test or the Fisher’s exact test (Kim 2017). Phi (φ) and risk ratio were used to describe the effect size of categorical data when there were two by two contingency tables (Kim 2017, Pautz, Olivier & Steyn 2018a). Cramer’s V was used to calculate effect size on larger tables (Kim 2017).

Continuous data were tested for normality (Online Appendix 1, Section 4). The normally distributed data are marked with a superscript ‘*n*’ in the results tables (Online Appendix 1, Section 4). Normally distributed data were analysed using unpaired Student’s *t*-tests and is presented as means and standard deviations. Data that were not normally distributed or were ranked data were analysed using the Mann–Whitney *U*-test and are presented as medians and interquartile ranges (IQRs). Effect sizes for data that underwent Student’s *t*-tests were calculated and are presented as Cohen’s (*d*_s_) (Pautz, Olivier & Steyn 2018b). Effect sizes for data that underwent the Mann– Whitney *U*-test are presented as probability of superiority (Pautz et al. 2018a). Effect sizes for continuous and ranked data were considered small if ≤ 0.2, moderate if ≥ 0.5, and large if ≥ 0.8 (Cohen 1992) (These interpretations of effect size were applied to both Cohen’s d and superiority probabilities). Effect sizes, *p*-values, confidence intervals and minimum clinically meaningful difference were taken into account when showing a difference between groups (Pautz et al. 2018a, 2018b).

Missing data were not replaced or estimated. If a participant declined to take part in a screening test or answered a question in the medical screening questionnaire, no data for that test were entered.

### Ethical considerations

The Human Research Ethics Committee of the University of the Witwatersrand granted Ethical Clearance (clearance certificate no.: M1611122). Our study was undertaken according to the ethical standards and guidelines of the Declaration of Helsinki. Identifiable information was removed from the dataset and study numbers were allocated to each participant. The link between the study numbers and identifiable information was kept in an access-controlled file to which only the first author had access.

Written informed consent was granted by participants over the age of 18 years old. Written informed assent was granted by participants under the age of 18 years old and informed written consent was granted by their legal guardians for them to take part in the study. An information sheet was provided to the parents or guardians by the Golf School of Excellence. Minors (participants under 18 years old) were only approached for inclusion in our study if their parents or legal guardian had given permission for them to take part in our study.

Minors were included as a high volume of sports participation has been shown to increase the risk of developing lower back pain in childhood and adolescence (Sato et al. 2011). Research involving minors is permitted if it is therapeutic research in the best interests of the minor (Strode & Slack 2015). The screening performed and analysis was similar to what they would typically undergo at the Golf School of Excellence. The inclusion of minors was approved by the University of the Witwatersrand’s Human Research Ethics Committee.

Please note that the identifiable photographs in the supplementary data do not include any research participants. These photographs are pictures of models who provided signed consent to have their photographs taken while demonstrating the screening exercises.

## Results

Forty-eight participants underwent the baseline assessment. Seven participants completed the screening, but were later lost to follow up. The total number of participants included in the final analysis was 41. The whole group’s median (interquartile range [IQR]) age was 19.80 (3) years; mean (standard deviation [s.d.]) body mass index (BMI) was 24.35 (3.68) kg/m^2^ and the median (IQR) handicap was 2 (3). Seventeen participants (41%) developed lower back pain. Fourteen golfers experienced one episode of lower back pain. Lower back pain episodes were measured in weeks. The first lower back pain episode median (IQR) duration was 2(4) weeks. Three participants reported two episodes of lower back pain (second episode mean [s.d.] duration was 2.3[0.56] weeks). The total length of lower back pain episodes (first and second) median (IQR) duration was 2(5) weeks. Three participants reported experiencing lower back pain and lower limb pain at the same time. Fifteen participants managed to continue to play golf while experiencing lower back pain.

It should also be observed that nine participants developed pain in their upper limb (upper limb injury incidence of 22%). Three of which were to the shoulder region and six in the elbow, wrist and hand region. Nine participants developed pain in their lower limb, three of which occurred in the hip region, two in the knee and four in the ankle (lower limb injury incidence of 22%). Five participants developed pain in the neck and thoracic region (neck and thoracic spine injury incidence of 12%).

Risk screening for lower back pain showed there was a difference between the group that developed lower back pain and the group that did not, in the following screening tests: rotational stability test non-dominant side (*p* = 0.01, effect size = 0.27), rotational stability test dominant side (*p* = 0.03; effect size = 0.29) and plank score (*p* = 0.03; effect size = 0.24) (Table 1). The lower back pain group performed better in rotational stability tests and in the plank test. There were no differences observed in any other screening tests for lower back pain (Online Appendix 1, Section 3 and Section 4.

**TABLE 1:** Table comparing ranking screening test results for those with lower back pain compared with those without lower back pain.

Variable	No lower back pain group percentage per category *n* = 24				Lower back pain group percentage per category *n* = 17				Comparison between no lower back pain and lower back pain group	
	Median	IQR	Lower CI	Upper CI	Median	IQR	Lower CI	Upper CI	*p*	effect size
Single leg squat non-dominant score	2	1	1.58	2.01	2	1	1.33	1.85	0.22	0.41
Single leg squat dominant score	2	1	1.58	2.01	2	1	1.34	1.96	0.36	0.43
Deep overhead squat score	2	1	2.1	2.49	2	1	2.15	2.67	0.43	0.44
Hurdle step non-dominant score	2	1	2.10	2.49	2	1	2.05	2.54	0.99	0.5
Hurdle step dominant score	2	1	2.10	2.49	2	1	2.1	2.61	0.68	0.47
In line lunge non-dominant score	2.5	1	2.28	2.72	3	1	2.26	2.79	0.86	0.49
In line lunge dominant score	3.5	1	2.28	2.72	3	1	2.26	2.79	0.86	0.49
Trunk stability push-up score	3	0	2.67	2.99	3	0	2.82	3.07	0.3	0.45
Rotational stability test non-dominant score	2	0	1.87	2.3	3	1	2.27	2.91	0.01	0.27
Rotational stability test dominant score	2	0	1.96	2.39	3	1	2.27	2.91	0.03	0.29
Plank score	3	4	2.41	4.72	7	4	4.38	7.22	0.03	0.24

IQR, interquartile range; CI, Confidence interval.

*n* = number of participants.

*p*-value calculated using Mann–Whitney *U* test.

### Confounding variables

The lower back pain group (median [IQR] = 3.33 [4.54] h/week) engaged in less range practice than the uninjured group (median [IQR] = 7.5 [8.5] h/week; *p* = 0.04; effect size = 0.31). The lower back pain group engaged in less cardiovascular training (median [IQR] = 1.25[0.96] h/week) than the uninjured group (median [IQR] = 1.67 [2.43] h/week; *p* = 0.04; effect size = 0.31). There were no other performance or training differences between the group that developed lower back pain and the group that did not develop back pain (Table 2). It should be observed that one participant’s FlightScope data did not record. It should also be noticed that one participant completed only 17 weeks of his training log and declined to complete any further training logs. He was, however, prepared to continue to be contacted for his injury monitoring. An average training for this participant was provided from 18 to 26 weeks.

**TABLE 2:** Comparison of performance and training variables for the lower back pain group versus the no lower back pain group.

Variable	No lower back pain group				Lower back pain group				Comparison between no lower back pain and lower back pain group	
	*n*	Mean (s.d.) or median (IQR)	95% CI lower	95% CI upper	*n*	Mean (s.d.) or median (IQR)	95% CI lower	95% CI upper	*p*	Effect size
Order of merit (rank)	17	24.00 (28)	16.87	32.54	11	20 (31)	14.28	36.45	0.96	0.49
Handicap (no)	24	1.5 (3)	1.34	3.99	17	2 (2.5)	1	3	0.76	0.47
Carry distance (m) †	24	214.51 (21.10)	205.61	223.41	16	218.07 (22.69)	205.98	230.16	0.62	0.16
Roll distance (m)	24	12.33 (11.58)	10.95	21.57	16	18.55 (22.20)	13.02	30.20	0.30	0.40
Total distance (m) †	24	230.73 (16.98)	223.56	237.9	16	239.81 (20.32)	228.81	250.46	0.14	0.49
Ball speed (km/h)	24	238.76 (13.71)	219.22	243.82	16	240.26 (22.07)	217.98	247.43	0.86	0.48
Club head speed (km/h)	24	177.30 (10.54)	164.61	182.51	16	175.32 (13.8)	160.77	181.23	0.33	0.41
Putting practice per week (h/week)	24	3.98 (6.74)	2.48	11.32	17	3.08 (3.12)	2.26	3.97	0.27	0.4
Short game practice per week (h/week)	24	4.1 (7.68)	2.56	11.83	17	2.94 (3.44)	2.12	4.07	0.13	0.36
Range practice per week (h/week)	24	7.5 (8.5)	5	14.2	17	3.33 (4.54)	2.5	7.37	0.04	0.31
Flexibility training per week (h/week)	24	1.17 (2.75)	0.49	4.98	17	0.92 (1.13)	0.69	1.44	0.37	0.42
Postural training per week (h/week)	24	0.7 (1.41)	−0.27	4.09	17	0.2 (0.42)	−0.22	1.69	0.32	0.41
Weight training per week (h/week)	24	2 (3.02)	1.37	6.43	17	1.81 (2.69)	0.93	2.4	0.34	0.41
Cardio per week (h/week)	24	1.67 (2.43)	1.28	4.57	17	1.25 (0.96)	0.78	1.59	0.04	0.31
Rounds of golf played per week	24	2.88 (2)	−11.83	45.09	17	3.04 (1.64)	1.82	3.32	0.58	0.45

Note: *p*-values calculated using independent t-test (normally distributed data) and Mann–Whitney *U* test (data not normally distributed). All other data were not normally distributed. Normally distributed data presented as means and s.d. Data not normally distributed are presented as medians and IQR.

IQR, interquartile range; s.d., standard deviation; CI, Confidence intervals.

*n* = number of participants CI were displayed as calculated, but please be advised that it is not possible to obtain a negative value for the confidence interval for these values, except for handicap. The lowest possible value is zero.

† , indicates that data were normally distributed.

The lower back pain group had a lower BMI and body mass than the uninjured group (BMI *p* = 0.04, body mass *p* = 0.02; BMI effect size = 0.68 body mass effect size = 0.78) (Table 3). There were no other differences between back pain groups in terms of baseline anthropometrics or medical history (Table 3 and Table 4).

**TABLE 3:** Comparison of baseline anthropometric data lower back pain group versus no lower back pain group.

Variable	No lower back pain group				Lower back pain group				Comparison between no lower back pain group and lower back pain group	
	*n*	Mean (s.d.) or Median (IQR)	95% CI lower	95% CI upper	*n*	Mean (s.d.) or Median (IQR)	95% CI lower	95% CI upper	*p*-value	Effect size
Age (years)†	24	19.54 (2.41)	18.53	20.56	17	20.18 (2.67)	18.8	21.55	0.43	0.25
Height (m)†	24	1.8 (0.08)	1.76	1.83	17	1.78 (0.05)	1.75	1.8	0.32	0.32
Body mass (kg)†	24	81.98 (12.54)	76.69	87.28	17	72.42 (11.79)	66.36	78.49	0.02	0.78
BMI (kg/m^2^)†	24	25.34 (3.53)	23.85	26.83	17	22.94 (3.52)	21.13	24.76	0.04	0.68

Note: *p*-values calculated using independent t-test (normally distributed data) and Mann–Whitney *U* test (data not normally distributed). All other data were not normally distributed. Normally distributed data presented as means and s.d. Data not normally distributed are presented as medians and IQR.

IQR, interquartile range; BMI, body mass index; s.d., standard deviation; CI, Confidence intervals.

*n* = number of participants.

† , indicates that data were normally distributed.

**TABLE 4:** Comparison of baseline medical history and history of injury: No lower back pain group versus the lower back pain group.

Variable	*n*	No lower back pain group with condition or health risk factor		Lower back pain group (*n*)	Lower back pain group with condition or health risk factor		*p*-value for between group difference at baseline	Between group Odds ratio	Odds ratio 95% CI lower	Odds ratio 95% CI upper	Risk ratio for lower back pain	Risk ratio 95% CI lower	Risk ratio 95% CI upper	Between group comparison effect size
		*n*	%		*n*	%								
Smoked at least 100 cigarettes in his lifetime	19	9	47.3	17	7	41.8	0.75	1.29	0.34	4.81	0.88	0.43	1.78	0.06
History vascular problems	19	1	5.26	16	1	6.25	1	0.83	0.05	14.48	1.1	0.26	4.62	0.02
History respiratory problems	17	5	29.41	16	3	18.75	0.69	1.81	0.35	9.24	0.72	0.27	1.9	0.12
History of cancer	19	1	5.26	16	1	6.25	1	0.83	0.05	14.48	1.1	0.26	4.62	0.02
History of musculoskeletal conditions	19	1	5.26	16	4	25	0.16	0.17	0.02	1.68	2	1.08	3.72	0.28
History of neurological conditions	19	3	15.79	16	2	12.5	1	1.31	0.19	9.02	0.86	0.27	2.68	0.05
History of gastrointestinal conditions	19	3	15.79	16	2	12.5	1	1.31	0.19	9.02	0.86	0.27	2.68	0.05
History of injury in past 6 months	18	7	38.89	16	12	75.0	0.05	0.21	0.48	0.93	2.37	0.96	5.87	0.36
History of back pain in the past 6 months	19	8	42.11	16	9	56.25	0.51	1.77	0.46	6.78	0.74	0.35	1.53	0.14

CI, Confidence intervals.

Data presented as number (*n*) per category and percentages.

*n* = number of participants.

*p*-value calculated using Pearson’s chi-squared test and Fisher’s exact test.

Although all the golfers were injury-free at the time of inclusion, 17 golfers had a history of lower back pain in the 6 months prior to inclusion. Thirteen golfers had experienced lower back pain in the month before taking part in our study. Five golfers had missed golf training days because of prior lower back pain (‘prior lower back pain’: back pain that occurred and resolved prior to enrolling in our study). Twenty-one training days had been missed in total because of prior lower back pain. Three golfers missed training because of prior lower back pain. Reasons given for prior lower back pain included: overtraining, S-shaped spine, swing faults, insidious onset, poor posture, carrying a golf bag, playing rugby, muscle spasm, bending, tiredness and high swing speed. The numerical pain score for prior back pain mean (s.d.) equaled 5(2) out of 10. Seven golfers had seen a physiotherapist for prior lower back pain. One golfer had been to see his general practitioner (GP) for his prior lower back pain. The golfers who had been seen by a physiotherapist or GP for prior lower back pain reported being diagnosed with poor vertebral alignment (physiotherapy diagnosis for one golfer) and muscle spasm. The golfers had received the following treatments for their prior lower back pain: RICE (rest, ice, compression, elevation techniques), massage, physiotherapy, strapping, anti-inflammatory topical plasters, strapping and foam rolling. There were, however, no differences at baseline between the two groups in terms of history of lower back pain (*p* = 0.51; effect size = 0.14; Table 4). There was, however, a possibility of an increased risk in developing lower back injury if the golfer had sustained any injury in the past 6 months. Twelve of the participants who developed lower back pain reported that they had an injury in the 6 months prior to inclusion, whereas only seven in the uninjured group reported any injury in the 6 months prior to inclusion (*p* = 0.05; effect size = 0.36).

It should be observed that the following tests had one participant who declined to perform them: pelvic tilt at set up posture (amount of motion), torso rotation test (quality of movement), toe touch test, 90/90 non-dominant tests in standing, lower quarter rotation dominant external rotation test, seated trunk rotation dominant side, straight leg raise test, oblique sit-up. This was because of time constraints. The screening took a long time to complete and some of the participants needed to leave because of transport arrangements. One of the participants’ FlightScope data did not record. Five participants declined to complete the medical history and injury history questionnaire. Some participants partly completed the medical history and injury history questionnaire (number of completed answers to each question is displayed in Table 4).

## Discussion

The lower back pain group scored better than their uninjured counterparts in three movement tests: rotational stability on the dominant and non-dominant sides and the plank test. The primary muscles used during these tests are the trunk muscles (Oliva-Lozano & Muyor 2020). These results would therefore imply that increased strength, endurance and control of the trunk musculature were a precursor to sustaining lower back pain. Strong spinal muscles may result in increased spinal muscle bracing via co-contraction movement strategies, which may result in increased spinal compression (Granata & Marras 1995) thereby increasing the risk of spinal injury. This may indicate that the golfers in the lower back pain group adopted a ‘tight control’ motor adaption (Van Dieën et al. 2019). This motor pattern may be observed in individuals with a history of lower back pain and involves increased trunk muscle co-contraction and subsequent reduced lumbar movement. Increased spinal muscle recruitment as a possible contributing factor to developing lower back pain in elite golfers is supported by a study conducted by Quinn et al. (2022) on 33 elite young male golfers, which showed that increased dominant *rectus abdominus* and dominant *latissimus dorsi* activation during the golf swing was associated with increased risk of developing lower back pain. It should be noticed that the effect sizes for this difference in rotational stability tests and plank test results between the low back pain group and the uninjured group were weak and therefore there is a risk of this finding having little clinical relevance.

It should also be observed that a large number of screening tests were conducted, which increases the risk of false positive conclusions (Weisstein 2021). The concept of this being a false positive is further supported by the lack of injury correlation in the other tests that assess spinal muscle strength and control, such as the trunk stability push-up, single leg bridge, pelvic tilt tests, pelvic rotation test, sit-ups and oblique sit-ups. Despite the possibility of a spurious result, according to our findings, improved spinal muscle strength and control may be a precursor to lower back pain in elite golfers, and therefore, future studies should attempt to confirm or negate these findings.

In terms of confounding variables related to training there were some differences between the back pain group and the no back pain group (cardio and range practice). It should be noticed that the effect sizes for these differences were weak and that all golfers practised at the same academy; therefore, the clinical relevance of these differences is unlikely to influence our findings. This common predictor was seen in the 12 participants who developed lower back pain and reported that they had an injury in the 6 months prior to inclusion, whereas only seven in the uninjured group reported any injury in the 6 months prior to inclusion (*p* = 0.05; effect size = 0.36). The effect of this confounding variable appears to have been small from a clinical relevance perspective. The *p*-values were not below 0.05 and the effect size was small. The authors carefully considered a prior history of lower back pain. Although all the golfers were injury free at inclusion, there were golfers in both the lower back pain group and the no lower back pain group who had complained of lower back pain in the 6 months prior to inclusion. There were, however, no differences between the two groups in terms of this variable at baseline assessment (*p* = 0.51; effect size = 0.14). A history of lower back pain did not appear to confound our results. This may have been because of application of inclusion criteria that excluded those with known serious spinal or hip pathology.

### Clinical implications

The golfers underwent 30 screening tests. Out of 30 screening tests, only three tests were associated with developing lower back pain and all these tests had a weak effect size. These tests are time-consuming and generally require that coaches require additional training to perform them, which has financial implications. Our study brings into question their usefulness in identifying players at risk of sustaining injury. This is consistent with findings in other sports. In a systematic review, Moran et al. (2017) concluded that there was moderate evidence against the use of the Functional Movement Screen in football to predict injury and that there was limited or conflicting evidence regarding its usefulness in predicting injury in college athletes, American football, ice hockey, basketball, running, fire fighters and police. A systematic review by Moore et al. (2019) provided more nuanced findings. They found mixed results regarding the Functional Movement Screen (FMS) composite score and subsequent injury risk. An FMS composite score below 15 and FMS asymmetry were associated with a small increase in injury in senior athletes but not in junior athletes. An FMS composite score below 15 was more likely to be associated with injury risk in rugby, American football and ice hockey than other sports. An FMS composite score below 15 was associated with a small increase in injury in males but not females. They also noticed that the effect sizes between the uninjured and injured group’s FMS composite scores were small and, therefore, may not be clinically meaningful. They concluded that FMS composite scores are not effective at identifying athletes at high risk of injury and should not be considered a complete injury-risk estimation tool (Moore et al. 2019). In contradiction to these reviews, a systematic review by Trinidad-Fernandez et al. (2019) concluded that because of the heterogenicity and varied definitions of injury that it is not possible to synthesise findings and draw any firm conclusion regarding FMS score and injury risk.

The questionable efficacy of screening tests to predict subsequent injury is a clear finding emerging from our data. Furthermore, it could be questioned whether we should continue to conduct screening tests in elite golfers. Although many of these tests do not seem to have a positive association with injury risk, they are useful because they give a comprehensive assessment of the golfers’ strength and range of motion (Bahr 2016). These tests have also been shown to be positively associated with golf performance and swing characteristics (Gulgin, Schulte & Crawley 2014; Speariett & Armstrong 2019). According to the International Olympic Committee (IOC), elite athletes should undergo comprehensive health evaluations (Bahr 2016; Ljungqvist et al. 2009). Comprehensive health evaluations are important for establishing rapport with the medical team, reviewing medication and preventing doping, establishing a performance baseline and to satisfy medico-legal requirements in some countries (Bahr 2016). Based on the findings of our study, we would recommend continuing to perform functional movement screening to establish rapport, determine baseline muscle function and length, but not as a screening tool to identify golfers at risk of lower back pain. The screening assessment may also allow for early identification of injury. Future research should confirm if the rotational stability or plank tests are useful predictors of lower back pain in young elite male golfers. These tests should ideally be conducted in isolation to exclude Bonferroni type errors.

## Limitations

FlightScope assessment was performed outdoors, where environmental conditions such as wind speed may have influenced measurements (even though many of the measurements are only made within the first few feet of ball flight). An outdoor setting was chosen above a laboratory setting as it was felt that this would reduce the anxiety that golfers (especially younger) may experience during the assessment while also being a more realistic representation of actual golfing performance.

A large number of screening tests were selected, which may have resulted in muscle fatigue and consequently may have influenced some of the test outcomes. The test participants were, however, elite golfers who are used to long training and practice sessions. The screening tests are also frequently performed as a battery of tests in clinical practice to screen for injury and not as isolated tests. This means that screening using a battery of tests is therefore more clinically relevant.

There was a difference in BMI and mass between the lower back pain group and the no lower back pain group. This difference had a moderate effect size and should be considered when interpreting our results. There is conflicting evidence surrounding the effect of body mass on developing back pain in golf (Smith et al. 2018). A systematic review by Smith et al. (2018) showed that golfers with lower back pain were heavier than golfers without lower back pain. In contradiction to these findings, a longitudinal study by Evans et al. (2005) on trainee professional golfers showed that golfers with a BMI below 25.7 kg/m^2^ were at greater risk of developing lower back pain. The impact of this variable (BMI) on the study results is therefore unknown. It is possible that heavier elite golfers had different swing biomechanics from lighter elite golfers (Evans et al. 2008), which may have showed their injury risk. A study conducted by Evans et al. (2008) show that the swing of golfers with greater BMI was not as susceptible to the fatigue effects of 40 min of putting as the lower BMI golfers were. They proposed that the increased body mass may have resulted in increased inertia during the swing, which helped to mitigate against the effects of fatigue. Future studies should confirm or negate these suggestions. These studies should ideally be performed in elite golfers and not in a mixed cohort including both elite and recreational golfers.

## Conclusion

Out of 30 movement screening tests, only three tests were associated with developing lower back pain. According to our study, movement screening is not useful to screen young elite male golfers at risk of developing lower back pain. Movement screening does, however, provide valuable information regarding elite athlete’s muscle function and length and as per the IOC recommendations it should continue to be used for this purpose.
